# Medical Jurisprudence

**Published:** 1892-03

**Authors:** Henry A. Riley

**Affiliations:** New York


### Progress in Medical Science.

### MEDICAL JURISPRUDENCE.

Conducted by HENRY A. RILEY, A. B., LL. B., New York.

### PHYSICAL EXAMINATION OF LITIGANTS.

THE Supreme Court of the United States recently decided that the Federal courts have no power to compel the plaintiff in an action for personal injuries to submit to a surgical examination, and the *Albany Law Journal* comments as follows:

It is difficult to accept the argument that a party may be compelled to produce particular evidence against his will, simply because he had the right to produce it if he wishes.

It seems clear to us that the true reason of the case has not been clearly propounded in either of the opinions, nor anywhere else, so far as we have read, excepting once. The court has no power to compel the suitor to produce any particular piece of evidence. He is suing for his own benefit, and may put in such evidence as he choose, taking upon himself the burden of satisfying the jury.

The jury may lean against him, because of his omission to produce certain available evidence, but the court has no more power to compel his exposure of his person to a surgical examination than to compel his production of a particular witness to the transaction in question, whom he omits or refuses to produce. This last class of omission is frequently commented on by opposing counsel as suspicious, but no one ever claimed that the party could be obliged to produce such evidence.

### BUFFALO AND ITS HEALTH DEPARTMENT.

THE revised charter of the City of Buffalo contains numerous provisions in regard to the Board of Health and its duties, some of which may be of interest to those who have not personally examined the new law.

The Board of Health consists of three members, the Mayor, the

President of the Board of Public Works, and the Health Commissioner.

The latter holds office for five years and must be a reputable and licensed physician, at least thirty years of age, and having an active experience as a practising physician of not less than five years.

The Health Commissioner has supervision over the care, removal, burial or incineration of the dead, the registration of births, marriages and deaths, of vital statistics, and with the approval of the Board, may make such rules and regulations as are necessary for the protection of the public health. The city is divided into eight health districts, for each of which a physician in good standing is to be appointed.

He is to have the title of city physician.

In addition there are to be two homeopathic physicians whose districts are to be designated by the Board.

It is the duty of the city physicians to render all necessary medical services to indigent sick persons in their districts, and report to the Board of Health all nuisances or unsanitary places, or any violations of health ordinances which may come under their notice.

The Board of Health has full power to abate nuisances, to take proper measures to prevent the entrance of pestilence or infectious diseases, and to stop persons coming from infected places and remove them to quarantine hospitals. It can disinfect buildings, vessels, clothing, etc., or destroy the same. The plans of all buildings to hold more than three families, of slaughter houses, livery stables, etc., etc., are to be submitted to the Board. The drainage and plumbing of public and private buildings must be according to plans approved by the Board. It is the duty of the Health Commissioner to visit all hospitals where patients supported by the city are received, at least once a month.

---

### THE GRIP AND THE LAW.

The English papers have just reported that the grip, or influenza, as it is termed in the cable dispatches, is so much of an epidemic that the authorities are attempting to stamp it out by a vigorous application of the laws relating to the public health.

These laws make it an offense for a person suffering from a contagious disease to visit public places. As the influenza seems to be regarded as a contagious disease, a number of persons have been arrested for violating the law, and have been fined $25 each.

It is said that this is the first instance in England of persons suffering from influenza being fined for endangering the public health by coming in contact with persons in the streets or public places.

It is not known that any attempt has been made in this country to suppress the grip by fines.

---

### IS ELECTRICITY MANUFACTURED ?

THERE has recently been a curious decision in Pennsylvania in reference to electricity.

The Northern Electric Light and Power Co. furnishes electricity for power or for illuminating and heating purposes to its customers, and it claimed to be a manufacturing company, so as to get the benefit of an act exempting the capital stock of such companies from taxation. The company, without question, generated electricity, stored it, disposed of it in quantities to suit purchasers, and was paid for so doing. It was claimed, however, that it did not manufacture electricity in the sense that term was used by the public and understood by the legislature at the time the act was passed in 1885, and the court adopted this view, holding the company liable to taxation.

